# Spontaneous bilateral renal pelvis rupture during CT in the absence of urinary tract obstruction: case report

**DOI:** 10.1186/s12894-020-00669-4

**Published:** 2020-07-13

**Authors:** Zongchen Li, Joey Chan Yiing Beh

**Affiliations:** 1 National University Hospital, Singapore, Singapore; 2 Ng Teng Fong General Hospital, Singapore, Singapore

**Keywords:** Spontaneous renal pelvis rupture, Forniceal rupture, Pyelosinus backflow, Contrast-induced osmotic diuresis, Case report

## Abstract

**Background:**

Atraumatic renal pelvis rupture without pre-existing renal or ureteric pathology is an uncommon event. It is reported in the setting of acute urinary tract obstruction, most often secondary to ureteric calculi. Typical symptoms include acute flank pain and nausea, mimicking pyelonephritis or other causes of acute abdomen. Spontaneous rupture occurring bilaterally without identifiable urinary tract obstruction is exceedingly rare, and has yet to be reported in current English literature. Possible contributing pathophysiological mechanisms can be postulated from reported cases of rupture with observed obstruction.

**Case presentation:**

A 58-year-old woman undergoing multiphasic computed tomography (CT) for evaluation of asymptomatic microscopic haematuria developed on-table bilateral renal pelvis rupture seen only after contrast administration, on the delayed phase. There was no significant past medical history of note. The patient remained asymptomatic throughout and after the study, and was managed conservatively. Follow-up radiographical imaging over a month showed resolution of urinoma and no further contrast extravasation. No complications or recurrence was subsequently noted.

**Conclusions:**

Spontaneous rupture of the renal pelvis can be a rare complication of intravenous contrast administration even in cases without identifiable urinary tract obstruction, and it can occur bilaterally. Cases can uncommonly be asymptomatic but typical symptoms should prompt evaluation of the kidneys, particularly when they are not included in the initial study or no delayed phase is protocolled. Interval imaging for resolution of urinoma and contrast extravasation is clinically relevant to monitor for and avoid infective sequelae.

## Background

Spontaneous renal pelvis rupture, also known as forniceal rupture or pyelosinus backflow, is an uncommon complication of acute urinary tract obstruction. In the context of intravenous urography during acute renal colic, Schwartz et al. defined a rupture as ‘spontaneous’ if it occurred in the absence of trauma, abdominal compression, destructive renal lesion or prior surgical and endoscopic intervention [1].

It is thought to occur as a result of rapidly increasing intraluminal pressure [2] presumably overwhelming slower reactive mechanisms that act to reduce urine output, as seen in chronic hydronephrosis. Typical symptoms mimic those of acute abdomen or pyelonephritis, with unilateral flank pain and nausea [3]. This event has been reported most frequently in association with obstructive ureteric calculi, with other aetiologies such as extrinsic ureteric compression or bladder outlet obstruction following distantly behind [4].

Asymptomatic spontaneous rupture occurring bilaterally in the absence of urinary tract obstruction is hence extremely unusual. To the best of the authors’ knowledge, this has not been described in current English literature. We report one such case precipitated by CT urography and discuss possible pathophysiological mechanisms.

## Case presentation

A 58-year-old woman with no significant past medical history was referred to the outpatient urology clinic for asymptomatic microscopic haematuria noted incidentally on urinalysis. Routine CT urography was performed in the pre-contrast, nephrographic and delayed phases. A total of 75 mL of iodinated contrast agent (300 mg I/mL concentration) was administered, and no diuretics were given.

**Fig. 1:**
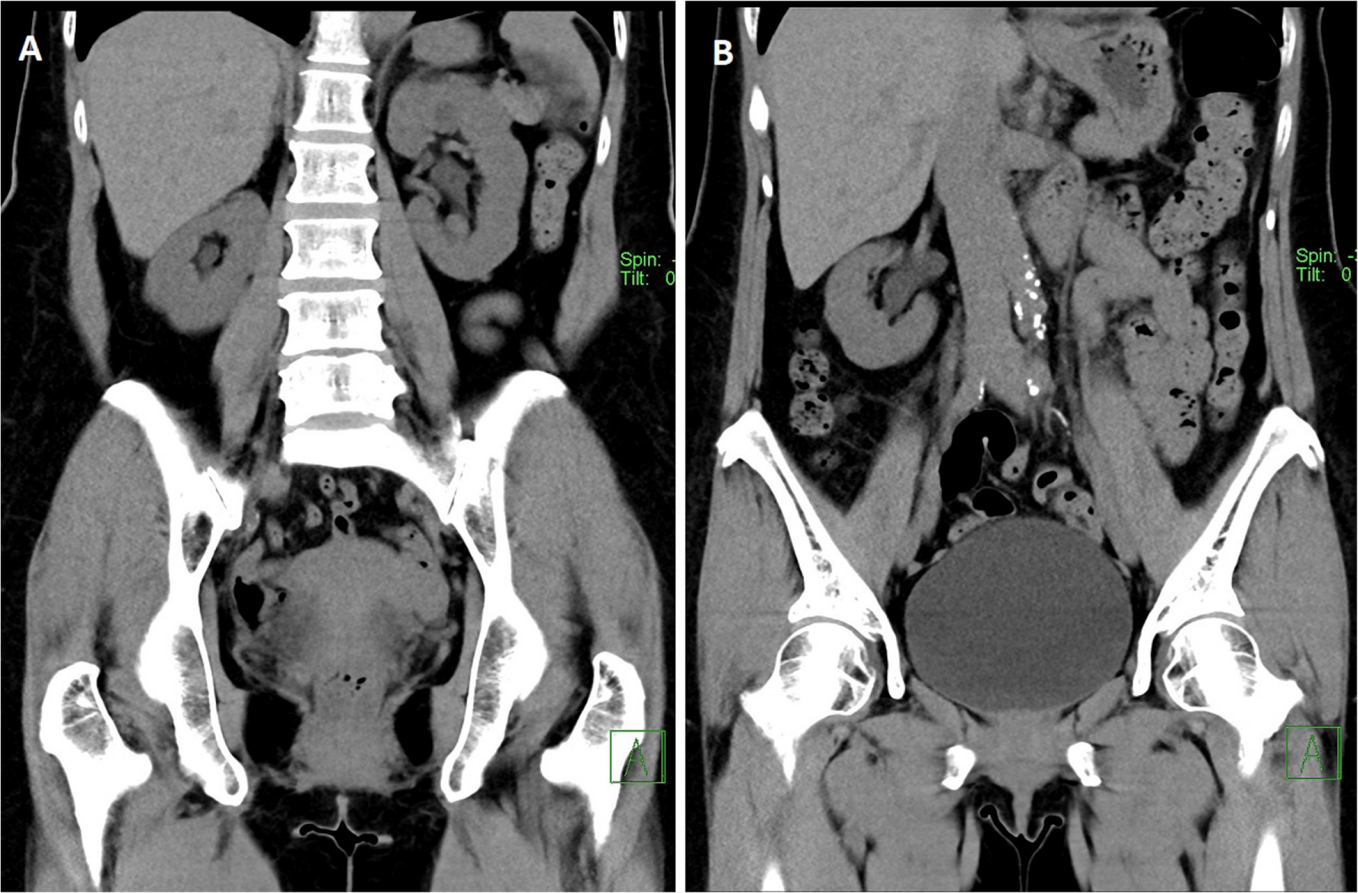
Non-contrast phase of the CT urogram showing no hydronephrosis or hydroureter bilaterally (**a**), and a well-distended urinary bladder (**b**). No ureteric or bladder calculus was seen

On the pre-contrast and nephrographic phases, a 2 mm non-obstructive renal calculus was noted on the right but the kidneys were otherwise unremarkable. No calculus was seen in the ureters and urinary bladder (Fig. 1). The latter was well-distended from voluntary retention of urine in preparation for the study. No perinephric fluid collection was noted at this point.

**Fig. 2:**
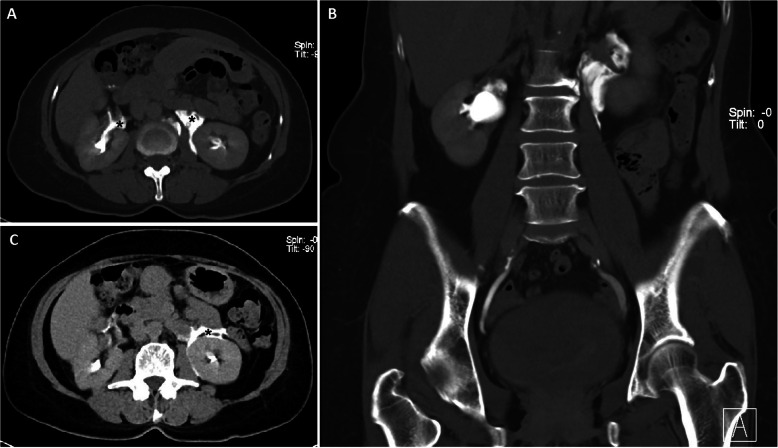
Initial delayed phase of the CT urogram (**a**, **b**) with peripelvic contrast extravasation marked with asterisks. Further delayed phase performed at 50 min (**c**) showing tracking of contrast

On the excretory phase performed at 6 min 13 s post-contrast, peripelvic contrast extravasation was observed bilaterally - suggestive of acute rupture (Fig. 2). No other site of urinoma was observed. A further delayed phase was performed at 50 min post-contrast to evaluate the extent of urinoma, which showed tracking of contrast along the retroperitoneum without further peripelvic extravasation.

Throughout the study, no flank pain or other symptom was reported; the patient remained asymptomatic even after the event was observed acutely on-table. No abdominal or flank tenderness was elicited on examination.

**Fig. 3:**
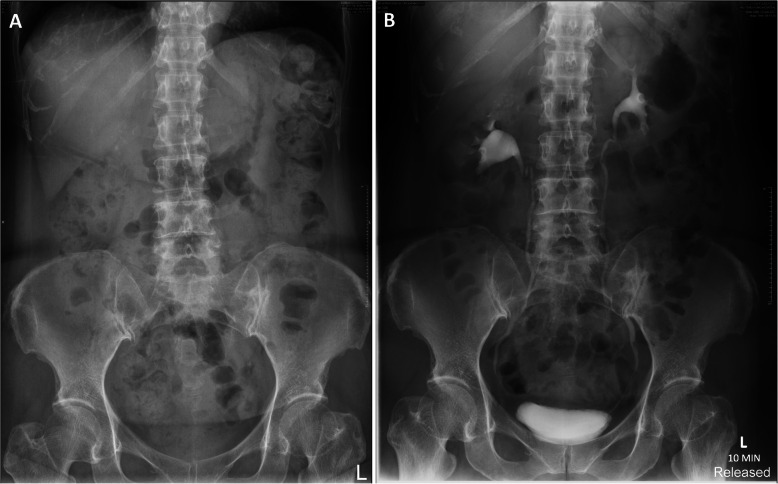
Interval plain radiograph performed the following day (**a**). Follow-up intravenous urography performed a month later (**b**). Both show no further contrast extravasation or persistent contrast pooling

She was managed conservatively and a radiograph performed the next day revealed no persistent contrast pooling. Follow-up intravenous urography performed a month after showed no recurrence of contrast extravasation (Fig. 3). Thereafter, she was discharged from follow-up after resolution of microscopic haematuria.

## Discussions and conclusions

This is a case demonstrating an exceedingly rare radiological event in the absence of classically associated risk factors. Reviewing related case reports, we discuss possible pathophysiological mechanisms though these are difficult to prove with a single case alone.

Rupture is reported to occur when intra-pelvic pressures exceed 25 to 75 mmHg and is seen most frequently at the fornices [5], possibly where the walls are the thinnest. Iodinated contrast agents are potent osmotic diuretics, and their role in precipitating rupture during an acute obstruction is clear. An early prospective study by Bernardino et al. demonstrated a positive relationship between the administered dose of intravenous contrast and the incidence of peripelvic contrast extravasation in patients with acute renal colic [2].

However, osmotic diuresis alone is an insufficient factor in the absence of obstruction. The primary mechanism is nonetheless likely dependent on this diuretic effect, given the temporal relationship between contrast administration and onset.

Unilateral renal pelvis rupture in the absence of obstruction has been described in the emergency care setting. Patients present first with flank pain, with a following CT demonstrating rupture where no obstruction is identified. Eken et al. suggest that unwitnessed transient obstruction, such as from a passed calculus, may be the most plausible mechanism [6]. Though that reported case had no known history of urinary calculi, another reported by Pampana et al. did [5].

The bilateral nature of rupture in this case makes transient obstruction by a passing ureteric calculus less probable. Moreover, a calculus was only demonstrated on one side prior to contrast administration. Transient obstruction more distally can affect both kidneys and remains a possible mechanism.

One other report of bilateral renal pelvis rupture occurring during contrast-enhanced CT was described by Chien et al. [7]. The case occurred during CT venography of the iliac veins, where a total of 170 mL of intravenous contrast was administered and post-rupture catheterization drained a urine volume in excess of 1 l. A background of bladder outlet obstruction from prostatomegaly was reported, and the authors attribute it as the cause of rupture. Acute flank pain during the scan first alerted radiological staff of this complication.

Notably, the case we report did not manifest flank pain. There was also no known history or symptom of urinary obstruction, though the bladder did appear distended on CT. It is plausible that voluntary urinary retention acted as a form of transient ‘obstruction’ at the bladder outlet.

Even so, majority of patients with a filled bladder do not develop this complication. The initial presentation of microscopic haematuria in this specific case may hence be relevant. Urinary calculi predispose to urinary tract rupture, with one postulated mechanism of urothelial injury during passage [8]. As no urothelial mass was identified in this case, the cause of haematuria could have been recent asymptomatic passage of calculi. We postulate that this could have additionally predisposed to rupture.

In the case reported by Chien et al., the patient was also treated conservatively. Interval imaging and urinalysis showed resolution of both microscopic haematuria and contrast extravasation. Serial imaging is of value, as large or persistent urinomas carry a risk of infection and loss of renal function [9]. Other modalities such as sonography can be considered as well [5]. Existing literature suggests that such a conservative approach is appropriate in the absence of complications (e.g. superimposed infection, kidney injury), risk factors for complications (e.g. sizeable urinoma 100 mL or larger) or a solitary kidney [10–12]. The detection of further contrast extravasation or unresolving urinoma in this case would have prompted consideration of drainage and urinary diversion.

In conclusion, spontaneous rupture of the renal pelvis is a potential (albeit rare) complication of intravenous contrast administration in the absence of urinary tract obstruction, and it can occur bilaterally. Although cases can uncommonly be asymptomatic, typical symptoms such as flank pain following contrast injection behoove physicians to evaluate the kidneys – particularly when they are not included in the initial study or no delayed phase is protocolled. A missed or untreated complication (i.e. large urinoma), could become a nidus for intra-abdominal infection and damage the urinary tract by fibrosis and granuloma formation. Rupture should also prompt physicians to actively evaluate for underlying causes. Interval follow-up imaging for resolution is clinically relevant to monitor for and avoid such infective sequelae through radiological-guided drainage or urological intervention.

## Data Availability

Not applicable.

## Abbreviation

CT: Computed tomography
